# Reduced Virulence of an Introduced Forest Pathogen over 50 Years

**DOI:** 10.3390/microorganisms7100420

**Published:** 2019-10-05

**Authors:** Rosie E. Bradshaw, Shannon Ormond, Pierre-Yves Dupont, Pranav Chettri, I. Kutay Ozturk, Rebecca L. McDougal, Lindsay S. Bulman, Murray P. Cox

**Affiliations:** 1 School of Fundamental Sciences and Bio-Protection Research Centre, Massey University, Palmerston North 4410, New Zealand; 2 Institute of Environmental Science and Research, Christchurch 8041, New Zealand; 3 AgResearch, Palmerston North 4410, New Zealand; 4 Scion, NZ Forest Research Institute Ltd, Rotorua 3010, New Zealand

**Keywords:** Dothistroma needle blight, forest pathogen, clonal population, virulence, mycotoxin

## Abstract

Pathogen incursions are a major impediment for global forest health. How pathogens and forest trees coexist over time, without pathogens simply killing their long-lived hosts, is a critical but unanswered question. The Dothistroma Needle Blight pathogen *Dothistroma septosporum* was introduced into New Zealand in the 1960s and remains a low-diversity, asexual population, providing a unique opportunity to analyze the evolution of a forest pathogen. Isolates of *D. septosporum* collected from commercial pine forests over 50 years were compared at whole-genome and phenotype levels. Limited genome diversity and increased diversification among recent isolates support the premise of a single introduction event. Isolates from the 1960s show significantly elevated virulence against *Pinus radiata* seedlings and produce higher levels of the virulence factor dothistromin compared to isolates collected in the 1990s and 2000s. However, later isolates have no increased tolerance to copper, used in fungicide treatments of infested forests and traditionally assumed to be a strong selection pressure. The isolated New Zealand population of this forest pathogen therefore appears to have become less virulent over time, likely in part to maintain the viability of its long-lived host. This finding has broad implications for forest health and highlights the benefits of long-term pathogen surveys.

## 1. Introduction

Healthy forests are critically important for the health of the planet. People depend on trees for provision of food, construction materials, and landscaping. In addition, sequestration of carbon by forest trees is vital for mitigating climate change [1,2]. Over their long lives, forest trees have to contend with many biotic and abiotic challenges that threaten their health. In the last two decades, forest disease epidemics have become more frequent and anthropogenic impacts, including climate change, have been implicated in these outbreaks [3,4,5,6,7]. The increase in global trade has facilitated the spread of pathogens into new locations, while changing land-use practices and intensive commercial forestry regimes have impacted on the severity of diseases and even awakened latent pathogens [8,9].

Long-term studies are needed to determine how plant pathogens evolve over time, and such studies are particularly important for managing the wellbeing of forests newly invaded by these pathogens. In agricultural systems, most crops are annual and rapid breeding for resistance, coupled with intensive fungicide treatments, can mitigate the effects of pathogens. But in forest pathosystems, the tree hosts live for decades, while the life cycles of pathogens are generally measured in weeks or months. Although breeding for resistance, and silvicultural and chemical controls, can be applied in some forest settings [10], there is a concern that genetic changes could occur in the pathogens that result in increased virulence [8,11], as is commonly observed with pathogens of annual crop plants [12]. These pathogen adaptations are more serious in long-lived species, such as forest trees, where there is limited capacity for genetic adaptation that would equip them to compete in an arms race with fast-evolving pathogens. Long-term studies of pathogen mutation and evolution are therefore necessary to help predict and inform forest management practices.

Long-term, often historical, studies of interactions between plants and their pathogens can yield helpful insights [13]. A long-term study examined fungal pathogens of wheat from archived samples collected over a 160-year period at Rothamsted in the United Kingdom [14]. The study showed changes in the relative abundance of two fungal wheat pathogens, now called *Parastagonospora nodorum* and *Zymoseptoria graminicola*, that correlated with levels of environmental pollution [14]. Some long-term studies of forest health include over 30 years of observations of *Pinus monticola* (Western white pine), which revealed the breakdown of major gene resistance to white pine blister rust due to the appearance of new races of the *Cronartium ribicola* pathogen [15]. In another analysis, long-term weather patterns of increased minimum temperatures and increased precipitation were shown to be associated with outbreaks of Dothistroma needle blight in British Columbia, Canada [16]. Studies of the North American fungal pathogen *Heterobasidion irregulare*, introduced into Italy during World War II, revealed the dynamics of its invasion, its hybridization with the native *H. annosum* and a high rate of gene flow from the native to the invading species, highlighting the importance of pathogen interactions in forest health [17,18,19]. However, few long-term studies of forest pathogens exist. Even where they do, regional and global gene flow, and high rates of recombination with incoming strains, can confound interpretation.

The New Zealand (NZ) population of *Dothistroma septosporum* (Dorogin) M. Morelet, the causal agent of Dothistroma Needle Blight (DNB), provides a unique opportunity to study change through time in an introduced forest pathogen within a semi-natural setting, controlled for many confounders. DNB is one of the worst foliar diseases of pine trees worldwide and has a global distribution [20]. In recent years, epidemics of DNB have occurred in the Northern hemisphere in native as well as commercial forests [20]. While many Northern hemisphere populations of *D. septosporum* show high genetic variability, the New Zealand population is globally unique because it is essentially clonal. *D. septosporum* is thought to be heterothallic with two known mating types [16], but there is no sexual recombination and only one mating type (MAT1-2) in the New Zealand *D. septosporum* population [21,22]. The pathogen is thought to have arrived in New Zealand in the late 1950s or early 1960s. Despite considerable surveillance, no new incursions have been detected since then [22], probably as a consequence of the geographic isolation of New Zealand and very strict biosecurity regulations. The low diversity of the NZ isolates has been demonstrated using three types of molecular genetic markers [22,23], and a preliminary comparison of two *D. septosporum* isolates collected in New Zealand in 1965 and 2005 revealed their genome sequences to be 99.99% identical [24].

Using a retrospective approach with historical samples of *D. septosporum* collected from New Zealand forests over a period of nearly 50 years, we explored whether genomic data support the hypothesis that a single genotype of *D. septosporum* was introduced into New Zealand. We then used these data to estimate a mutation rate for *D. septosporum* in the commercial forest environment. The genomic data were augmented by a range of phenotypic data, including pathogen virulence and tolerance to copper fungicide, to look for signatures indicative of adaptation. 

## 2. Materials and Methods

### 2.1. New Zealand Dothistroma septosporum Isolates, Growth, and Dothistromin Assays

*D. septosporum* isolates (Table 1) were wild type (WT) forest isolates collected from pine needles over a 50-year period and stored at Scion (New Zealand Forest Research) as agar plugs in vials of distilled water at 4 °C or under oil. Over the period of storage, these isolates were periodically grown on 2% malt extract and re-stored to maintain viability and to check for the absence of bacterial contamination. Older isolates were originally named as *Dothistroma pini* and referred to as such in the literature prior to a taxonomic revision [25], but all *Dothistroma* isolates in New Zealand are now known to be *D. septosporum* [22]. 

Insufficient isolates were available from the same region of the South Island as the 2005 NZE10 genome reference strain [26], so all isolates in this study derive from the central region of the North island of New Zealand, with most isolated from *Pinus radiata* D. Don. The collection included four of the oldest viable isolates (all collected in the 1960s), four isolates collected between 2006 and 2013, and two with intermediate collection dates (1991 and 1994); we refer to these sets of isolates as “decade groups”. The NZE10 reference strain was included in the 2000s decade group unless otherwise noted. Two pairs of isolates collected from the same region on the same day were included: 16N and 16P (collected on 1 September, 1969), and 2737 and 2738 (20 October, 2006) (Table 1).

Mycelium for DNA extraction was grown in 25 mL Dothistroma broth (DB; 5% malt extract, 2.8% nutrient broth) in 125 mL flasks at 22 °C for 7–10 d with shaking at 200 rpm. Samples were freeze-dried, ground, and the DNA extracted with a Plant Genomic DNA Mini Kit (Geneaid, New Taipei, Taiwan), and quantified using a Qubit Fluorometer (Waltham, MA, USA.).

For dothistromin quantification, 200 mg of freshly cultured *D. septosporum* mycelium of each isolate was macerated in 500 µL sterile water with a micropestle. Then 100 µL of the suspension was inoculated into 25 mL DB and grown as above, with continuous light. For each isolate, three biological replicates were analyzed, and the mycelium from each flask freeze-dried for dry weight determination. Dothistromin was extracted from culture broths by agitation for 72 h at room temperature with an equal volume of ethyl acetate, acidified with 0.1% formic acid, then quantified using HPLC (Dionex HPLC system, Sunnyvale, CA, USA) as described previously [27]. 

### 2.2. DNA Sequencing and Read Mapping

Library preparation (Illumina gDNA TruSeq) and sequencing (Illumina HiSeq2500 HT chemistry, 125 bp paired end) were carried out by the Australian Genome Research Facility Ltd (Brisbane, Australia). All libraries were sequenced on one Illumina HiSeq2500 lane. Sequencing adapter and primer sequences were removed from raw sequence data using fastq-mcf [28], then reads were quality trimmed (Phred score >20) using SolexaQA v3.1.4 [29] and trimmed reads <50 bases were discarded. General quality parameters of the data were assessed using FastQC v0.11.5 [30]. Reads were mapped to the reference genome of *D. septosporum* (New Zealand isolate NZE10; http://genome.jgi.doe.gov/Dotse1/) [26] with the Burrows–Wheeler Aligner v0.7.15-r1140 [31]. Bedtools v2.19.1 [32] was used to determine genome-wide read coverage and gene coverage based on annotated *D. septosporum* loci (entire coding sequence, including introns; http://genome.jgi.doe.gov/Dotse1/). Sequence reads have been deposited in the Sequence Read Archive (SRA; http://www.ncbi.nlm.nih.gov/sra/) as BioProject PRJNA426106.

### 2.3. Single Nucleotide Polymorphism Analysis

Trimmed reads were mapped to the *D. septosporum* NZE10 genome using methods described earlier [33], except using the tophat v2.2.1 mapper [34]. Mapped reads were analysed using freebayes v1.1.0-46 [35] with ploidy set to 1 (haploid) to detect variants between each of the samples and the NZE10 reference. The resulting VCF files were annotated and the potential effects of single nucleotide polymorphisms (SNPs) categorised based on the *D. septosporum* NZE10 filtered gene models using SnpEff v4.3t with quality filtering at Q ≥ 20 and a minimum of 5 mapped reads for each SNP [36]. The numbers of SNPs within genes were determined using the Bedtools [32] intersect command.

The individual SNPs were further analysed using Geneious v9.1.8 [37]. Possible deletions and duplications, compared to the NZE10 reference, were analysed manually using the read-mapping visualization functions of the IGV v2.4 browser [38] and Tablet v1.17.08.17 [39]. VCF files were filtered to remove any SNPs associated with indels, using the remove-indels command in VCFtools [40], and to remove SNPs in repetitive regions, based on the coordinates of known repeat regions greater than 200 bp (Table S5) using the VCF exclude-bed tool. SNP differences between each pair of isolates sequenced in this study were determined using vcf-compare in VCFtools [40].

A phylogenetic tree was constructed using all filtered SNPs (compared to NZE10) from the New Zealand isolates described here, along with those from genomes of *D. septosporum* isolates from Colombia (COL), Ecuador (ECU), Canada (CAN), Germany (ALP3), and Russia (RUS1) [24].

### 2.4. Mutation Rate Estimation

A mutation rate was determined by calculating the following in R v3.4.3 for each pairwise combination of isolates: (number of SNP differences/number of mapped bases)/time difference in years. The SNP differences between each pair of isolates were based on filtered SNPs (indels removed and repeat masked). The SNP differences were normalized by the average number of bases in the NZE10 reference genome to which the reads for that pair of isolates mapped; the read mapping was computed using the depth tool from SAMtools [41]. The time difference in years was based on differences in sample collection date (Table S2). Finally, an average mutation rate was determined based on pairwise rates calculated for (i) all 1960s isolates compared to all 2000s isolates and (ii) all 1960s isolates compared to all more recent isolates (1990s and 2000s).

### 2.5. Pathogenicity Assays

The virulence of the *D. septosporum* isolates was assessed using 1 y old *Pinus radiata* seedlings grown from a Dothistroma Needle Blight-susceptible seedlot, with four replicate trees per isolate. The isolates were grown for 9 d at 22 °C on PMMG agar (pine minimal medium glucose agar [42]) to produce spores for inoculum and the spore yields from three biological replicates were quantified using a cytometer. Each seedling was inoculated by spraying with approximately 5 ml of 5 × 10^6^ spores/mL, then incubated in one of four custom-built misting chambers (each containing one replicate seedling for each isolate) using methods described previously [43]. At 13 weeks post inoculation, needles with disease lesions were counted and harvested [43] and DNA was extracted from up to 34 lesions per plant, depending on availability. In some cases, lesions from two replicate plants had to be combined to obtain sufficient material, so the DNA analysis was therefore carried out with three replicates. Estimations of *D. septosporum* biomass were made using a real-time PCR assay targeting a *D. septosporum* gene (*DsPksA*) and a *P. radiata* gene (*CAD*) for reference, using methods described previously [27]. The fungal biomass was estimated by the amount of fungal DNA, normalized in two ways: (i) by lesion number and (ii) by mg dry weight of infected plant tissue (see Table S4).

### 2.6. Spore Germination, Growth, and Copper Tolerance Assays

Copper fungicide (as copper oxychloride or cuprous oxide) has been used in New Zealand forests for operational control of Dothistroma Needle Blight since the summer of 1965–1966 [44], although copper concentrations have been reduced over time as aerial spraying technologies have improved [10,45]. Because this is a potential selection pressure, we looked for evidence of increased tolerance to copper in the *D. septosporum* isolates from the 1960s to the present. 

As an indication of sporulation ability, we compared sporulation of the isolates on PMMG growth medium containing pine needle extracts [42]. Spores were harvested as for the pathogenicity assay and the spore yields counted with a cytometer. To assess spore germination, the spore concentrations were adjusted to 1 × 10^7^ spores/mL, then incubated for up to 48 h at ambient temperature (~20 °C) in sterile water or in 50 μg/mL CuSO_4_·5H_2_O (equivalent to approximately 12.7 μg/mL Cu^2+^ ions). Spore germination was recorded by counting at least 100 spores for each of three replicates for each isolate and recording numbers of germinated spores with germ tubes. For growth rate determination, plates containing Dothistroma media (DM) [46] and 0, 50, or 100 μg/mL CuSO_4_·5H_2_O were inoculated with three separate 25 μL spots of ground mycelium of *D. septosporum* per plate (approximately 5 mm^2^ of mycelium from DM agar plates per spot) and incubated at 22 °C. Measurements of colony diameters (*n* = 6) were taken and growth rates calculated as mm/d over 26 d when the colonies were still in growth phase.

### 2.7. Statistical Analysis

All statistics were performed in R v3.5.1 [47]. Binomial probabilities and nested (“hierarchical”) ANOVAs were calculated in base R. In our design, the first level of the ANOVA is the decade group, within which sit the isolates (second level), each of which has replicate values of the measured variables. The unbiased effect size estimator ω^2^ [48] was calculated using sjstats v0.17.2 [49] and mixed linear models, used for confirmation, were calculated using nlme v3.1-137 [50]. Statistical analyses were performed to test the null hypothesis that there was no effect of decade group on phenotype, and included NZE10 as well as all ten resequenced isolates. Isolates were analysed in three decade groups: 1960s (16F, 16G, 16N, 16P), 1990s (16R, 16V), and 2000s (NZE10, 2737, 2738, 3287, 3769), as well as in two decade groups (1960s and 2000s only). 

## 3. Results

### 3.1. Genome Sequencing and Single Nucleotide Polymorphisms of D. septosporum Isolates

Between 5.8 and 6.7 Gb of paired-end DNA sequence read data were obtained per isolate and over 93% of reads for each isolate mapped to the NZE10 reference genome (Table 1). Single nucleotide polymorphism (SNP) variants were determined for each of the isolate genomes compared to the *D. septosporum* NZE10 reference genome. Although over 3000 SNPs were computed for each of these comparisons, about 90% of these variants fell into regions of repetitive sequence or sequences with putative insertions or deletions (indels), and manual inspection of SNP calls in these regions suggested that they were unreliable. When repeat and indel features were masked in the genomes, 227–424 reliable filtered SNPs were identified (Table 1), of which 37–51 were nonsynonymous SNPs located in annotated coding sequences (Table S1). 

### 3.2. SNP Differences Reveal Increased Diversity between Isolates over Time 

**Figure 1:**
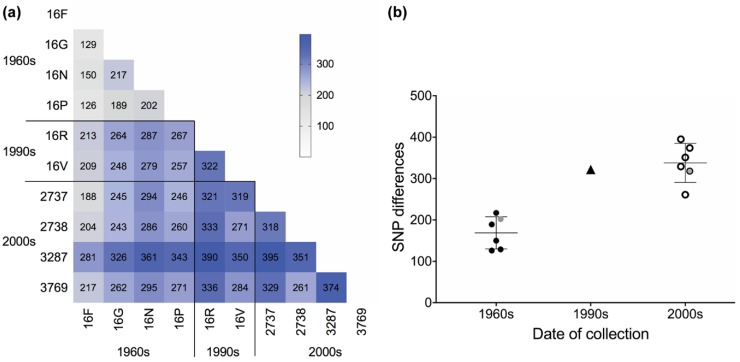
SNP differences between isolates of *Dothistroma septosporum.* (**a**) Heatmap of whole-genome filtered SNP differences between pairs of isolates of *D. septosporum*. (**b**) Decade-group comparisons of pairwise filtered SNP differences between isolates of *D. septosporum* from the 1960s (16F–16P), 1990s (16R and 16V) and 2000s (2737–3769). The pairwise comparisons between isolates collected on the same day (1960s 16N/16P and 2000s 2737/2738) are indicated by grey data points.

The ten re-sequenced isolates were analysed using pairwise comparisons of SNPs. A heatmap of pairwise SNP sharing (Figure 1a) shows close similarity of the four 1960s isolates, in contrast to more divergent comparisons with all later isolates. The data further suggest that the 3287 isolate, collected in 2007, had the highest number of SNP differences compared to those of all other isolates, (mean of 352 SNP differences or 1.2 × 10^−3^% of the genome) while the oldest isolate (16F) had the fewest (mean of 191 SNP differences or 6.3 × 10^−4^% of the genome).

We considered whether the data support the hypothesis that a single genotype of *D. septosporum* was introduced into New Zealand in the 1960s. New Zealand isolates of *D. septosporum* are known to be clonal based on their low genetic diversity [22] and presence of only a single mating type [21,22]. This hypothesis leads to a prediction that lower diversity should be observed among the genomes of older isolates, with greater divergence among more recent isolates as mutations have accumulated over time. Pairwise comparisons showed that the number of observed SNP differences doubled from the 1960s (range 126–217) to the 2000s (range 261–395), with this change appearing to be largely in place by the 1990s (one pairwise value = 322). This increase in SNP numbers is statistically significant (F_2,8_ = 20.15, *p* = 0.00075 ***, ω^2^ = 0.78), with change between the decade periods explaining nearly 80% of the variance observed in the SNP counts (Figure 1b). The same effect held when the analysis was repeated without the single 1990s pairwise comparison point (effect size ω^2^ of 0.79 vs. 0.78 with the 1990s value included).

These results were concordant with the closer similarity of the 1960s isolates based on actual SNP sharing (Figure 1a). They also occurred despite two of the 1960s isolates being collected from different host species (Table 1), while all later isolates were collected from a single host species (*Pinus radiata*). The pair of 1960s isolates (16N and 16P) that were collected on the same day in the same region were no more similar to each other (202 SNP differences) than other pairwise combinations of isolates collected in the same decade (range 126–217 SNP differences). Although there were some genetic differences among the New Zealand 1960s isolates, they were much more similar to each other than between the highly diverse global population of *D. septosporum* (Figure S1) [24].

### 3.3. Estimating Mutation Rates

The availability of SNP data for clonal isolates of *D. septosporum* collected on specific dates over several decades in the New Zealand forest environment enabled us to estimate a mutation rate. Based on SNP differences to NZE10, an estimate of mutation rate per year was made between each pair of isolates (Table S2). Combining all pairwise comparisons between the 1960s and the 2000s isolates, the mutation rate was estimated as 2.23 × 10^−7^ ± 0.48 × 10^−7^ SNPs per nucleotide per year (mean ± SD). A similar rate was obtained when the 1990s isolates were included as well (Table S2). Comparisons between isolates within each of the decade groups showed higher rates of genetic change (ranging from 1.08 × 10^−6^ to 1.18 × 10^−5^ SNPs per nucleotide per year, Table S2). These higher rates are concordant with the expected diversification of isolates over time. 

### 3.4. Dothistromin Production and Virulence Declined Significantly over Time

**Figure 2:**
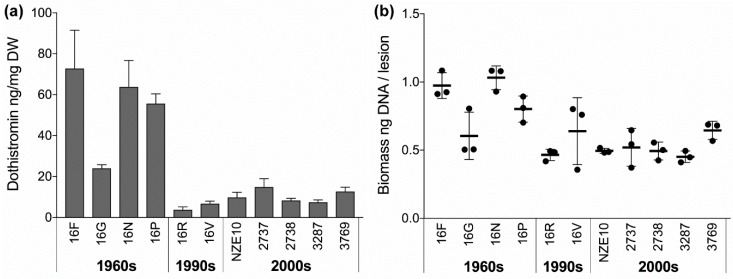
Dothistromin production and virulence assessments of *Dothistroma septosporum* isolates. (**a**) Levels of dothistromin produced in culture by the ten re-sequenced *D. septosporum* isolates, as well as the reference isolate NZE10. Values are normalised by dry weight of fungal biomass and shown as mean and SD of three biological replicates. Nested ANOVA and effect size analysis showed a significant effect of decade group on dothistromin levels (*p* = 0.002, *ω*^2^ = 0.74). (**b**) Virulence assessment of the same set of isolates of *D. septosporum* shown as relative fungal biomass per disease lesion on *Pinus radiata* needles. Biomass was determined by qPCR by its proxy, *D. septosporum* DNA levels, and is shown with mean and SD of three biological replicates. Nested ANOVA and effect size analysis showed a significant effect of decade group on biomass levels (*p* = 0.016, *ω*^2^ = 0.50).

The isolates were assessed to determine whether changes in phenotype occurred over 50 years in the forest environment. The mycotoxin dothistromin is the primary virulence factor in Dothistroma Needle Blight and is required for lesion expansion [51]. It is produced and secreted in culture, with levels of production known to vary between global isolates of *D. septosporum* [46]. We therefore assessed the ability of the New Zealand isolates to produce dothistromin in culture. The four 1960s isolates produced, on average, six-fold more dothistromin than isolates collected at later time points (Figure 2a), although isolate 16G produced notably less than the other three isolates in the 1960s group. A strong time effect on dothistromin levels was observed when all three decade groups were compared (F_2,8_ = 14.87, *p* = 0.0020 **, ω^2^ = 0.74), with these time periods accounting for 74% of the variance seen in dothistromin values. This indicates a strong dependence of this phenotype on the collection date of the isolates (Table 2). Similar results were obtained when the small number of 1990s values were excluded, and only the 1960s and 2000s decade groups were compared (Table S3). An additional isolate (4554), collected in 2017, was also assessed for dothistromin production in the same way and produced 7.2 ± 1.4 ng dothistromin/mg dry weight (DW), within the range of other isolates collected in the 2000s.

Because the 1960s isolates produced higher levels of dothistromin in culture, we tested whether they had increased virulence compared to the more recent isolates in planta, using pathogen biomass production in disease lesions as a measure of virulence. *Pinus radiata* seedlings inoculated with spores from each of the isolates were incubated under controlled laboratory conditions and disease lesions harvested after 14 weeks. Estimates of *D. septosporum* biomass, based on pathogen DNA levels revealed by species-specific qPCR, revealed significantly higher biomass levels (per lesion or per mg dry weight of lesion) in the 1960s group, compared to more recent isolates (Figure 2b, Table 2). With the exception of isolate 16G, the 1960s isolates showed increased virulence compared to the 1990s and 2000s isolates. 

Due to the small numbers and sizes of lesions, it was not possible to quantify dothistromin production per lesion in planta. However, there was a clear linear correlation between levels of dothistromin produced in culture and *D. septosporum* biomass per lesion when analysing all values from all isolates (*r* = 0.84, *r*^2^ = 0.71, *p* = 9.92 × 10^−10^). Tellingly, the lower in planta biomass of isolate 16G mirrors its lower production of dothistromin compared to the other isolates from the 1960s (Figure 2). Because the virulence of dothistromin is so tightly associated with increases in lesion size and fungal biomass [51], these results provide the first indication that dothistromin levels in culture may accurately reflect dothistromin activity in planta, at least under controlled conditions.

### 3.5. Sporulation, Growth, and the Effects of Copper

*D. septosporum* relies exclusively on the production of asexual spores for its dissemination in New Zealand forests because there is only one mating type present in the population [21,22]. Sporulation was highly variable between the isolates, but no significant effect of decade of collection was observed (Table 2, Tables S3 and S4). Spore germination rates were also variable, although a small but significant effect of decade group was seen (F_2,8_ = 7.71, *p* = 0.014^*^, ω^2^ = 0.14), with a tendency for the 1960s isolates to have lower rates of germination than more recent isolates (mean of 35.8% for 1960s vs. 44.5% for 2000s, Table 2, Tables S3 and S4).

Spore germination in the presence of copper (50 μg/mL CuSO_4_·5H_2_O) was reduced to approximately 49% of germination, relative to the 0 μg/mL copper baseline, for the 1960s isolates, and correspondingly reduced to 32% and 43% for the 1990s and 2000s isolates, respectively. This is opposite to what would be expected if the more recent isolates had increased copper tolerance. Moreover, the variability in spore germination between isolates was high, and differences in the effects of copper between decade groups were not significant (Table 2), suggesting that the isolates have no time related differences in tolerance to copper. 

Growth rates of the isolates in culture were compared in two independent experiments. The mean radial growth rates of the 1960s isolates were slower than those for later isolates by 12.8% (experiment 1) and 10.9% (experiment 2) (Table 2), but these lower growth rates were not statistically significant. The effect of copper on growth rates was then tested at two copper concentrations. In the presence of 50 μg/mL CuSO_4_·5H_2_O, the 1960s isolates grew almost as well as on media without copper (growth ratio of 0.93), while the more recent isolates were significantly more affected by copper (growth ratios of 0.79 and 0.80) (Table 2), which is opposite to what would be expected if adaptation for increased copper tolerance had occurred. With 100 μg/mL CuSO_4_·5H_2_O, a small but significantly higher growth rate of recent isolates compared to 1960s isolates was observed, but when normalized by growth rates in the absence of copper (i.e., as a growth ratio), these differences were not significant (Table 2). Taken together, these results provide no convincing evidence for increased tolerance to copper over time.

### 3.6. Are Specific Genetic Changes Associated with Differences in Phenotypes?

Because isolates from different decades showed significant variation in dothistromin levels, we first looked for genetic changes in known dothistromin genes. Twenty genes within six loci on chromosome 12 are known to be involved in dothistromin biosynthesis [52]. Amongst the isolates studied, no variation was found in the sequences of these genes or within 1 kb of sequence flanking them with one exception: isolate 2738 had a unique SNP at position 88486, approximately 250 bases upstream of *DsDotC* (JGI gene ID 75413). Although DsDotC facilitates dothistromin production [53], the functional importance of this specific upstream change is not known and, in any case, is restricted to a single isolate and so cannot explain the striking decade differences observed. Likewise, no sequence variation was found in, or within 1 kb of, other genes previously shown to be involved in the regulation of dothistromin biosynthesis (*DsVeA*, *LaeA*, *Kat2*, *Kmt1*, and *Kmt6*) [27,54,55]. 

Isolate 16F appears to carry a chromosomal duplication, with approximately twice the number of reads mapping to a large portion (76%) of NZE10 chromosome 11 relative to baseline levels across the rest of the genome (Figure S2a). This region includes genes speculated to be involved in increased dothistromin production by a very high dothistromin-producing German isolate of *D. septosporum*, ALP3, which also has a duplication of chromosome 11 along with two other chromosomes [24]. Interestingly, isolate 16F was the highest dothistromin producer of all New Zealand isolates in the current study. However, this change in a single isolate cannot explain the time period differences in virulence that we observed. Apart from this chromosome 11 duplication, the only other large-scale duplication or deletion noted was an approximately 1 kb deletion in chromosome 1 found in isolates 2738 and 3769, which fell within a hypothetical protein gene with unknown function (Ds68999) (Figure S2b).

Manual curation of SNPs within the coding regions of all genes revealed variants that were shared between two or more isolates. None of these variants (top section, Table 3) coincide with the decade groups, although they do reveal possible relationships between earlier and later isolates. In particular, isolates 16V, 2738, 3287, and 3769 share multiple SNPs, suggesting that 16V may be ancestral to a lineage with the three later isolates as descendants. Similarly, isolate 2737 shares multiple SNPs with 16F, 16N, and 16P, which may form another lineage. These two groups comprise a mixture of host species and collection locations (Table 1), providing no evidence that lineages are host-adaptive or geographically distinct. Furthermore, these data show gene differences between isolates collected on the same day in the same region (16N/16P and 2737/2738). Among the 1960s isolates, they also highlight genetic differences between the low dothistromin-producer 16G and the other 1960s isolates (16F, 16N, 16P), suggesting that 16G was an early, phenotypically different isolate that is not closely related to later low virulence isolates.

Genes with different SNPs in two or more isolates were also identified. Of these, genes known to be expressed by isolate NZE10 in planta [33] are shown in Table 3 (lower section). The numbers of polymorphisms observed in these genes are higher than expected given the genome wide mutation rate (binomial probability, all *p* ≪ 0.003), suggesting they may have been the target of selection. The three most highly expressed genes (71010, 72963, and 137952) are all predicted to have roles in transcription regulation or RNA processing. Another gene (74323), predicted to encode an RNA splicing coactivator, had six independent polymorphisms in different isolates (Table 3); five of these were frameshift or nonsense mutations that strongly suggest selection for loss of function by pseudogenization.

## 4. Discussion

### 4.1. Genome Diversity Suggests the Introduction of Several Closely-Related Isolates of D. septosporum

We tested the hypothesis that *D. septosporum* isolates in New Zealand originate from the introduction of a single genotype. *D. septosporum* is rumoured to have been introduced into New Zealand by forestry officials who visited East Africa in 1957 to observe Dothistroma Needle Blight. Regardless of whether this is true [22], five years later the pathogen was found in disease lesions on pines in the central North Island of New Zealand [56]. Some of those very early samples are included in this study.

A prediction from the single genotype introduction hypothesis, namely that recent isolates should show more diversity than 1960s isolates, was strongly supported by comparisons of SNP differences between isolates collected over a 50-year period from the same general region of New Zealand as the first disease reports. However, the level of diversity between the four 1960s isolates, although low, is more than might be expected if only a single isolate had been introduced. Some of this observed diversity may have arisen over time in culture and storage, or in the forest between the time of arrival and the time of collection. However, part of the diversity more likely already existed in the isolates that were carried into New Zealand. Because the diversity of the 1960s isolates was about half of the diversity seen in the 2000s isolates (a time span of 40 years), it could be argued that a single isolate was introduced into New Zealand around 40 years before the 1960s (i.e., in the 1920s) and mutated over time to produce the diversity seen in the 1960s samples. However, historical evidence supports the late 1950s/early 1960s as the most likely time when *D. septosporum* was introduced into New Zealand and, despite regular surveying, there are no reports of the disease any earlier than the 1960s. A thorough compilation of worldwide first disease reports [20] suggests that while Dothistroma Needle Blight was in parts of Europe in 1907 and the USA in 1917, it was not generally found in the Southern hemisphere until the 1960s, apart from an isolated report from Zimbabwe in 1943. This evidence therefore favours the arrival of *D. septosporum* in New Zealand in the late 1950s or early 1960s, but in the form of several very closely related isolates with genetic diversity similar to that reported for the 1960s isolates here. Indeed, because different genotypes can occur in a contaminated plant sample, introduction of several genotypes is more likely than a single genotype even if only one sample was the source of invasion. 

### 4.2. Estimation of a Mutation Rate 

Regardless, New Zealand *D. septosporum* isolates are still strikingly clonal when viewed against the backdrop of global diversity (Figure S1) [24]. Together with their asexual nature in New Zealand due to the presence of only one mating type, this provided a unique opportunity to estimate a rate of mutation for the pathogen in a non-laboratory environment using genome-wide data. The estimated mutation rate of 2.23 × 10^−7^ ± 0.48 × 10^−7^ SNPs per nucleotide per year is high compared to rates reported in other studies, although the parameters of those studies were often quite different than the one undertaken here. Rates of 0.9 × 10^−9^ to 16.7 × 10^−9^ mutations per nucleotide per year were calculated for neutral (synonymous) mutations in protein coding genes of Eurotiomycetes [57], and a genome-wide single nucleotide mutation rate of 1.67 ± 0.04 × 10^−10^ per nucleotide per generation was calculated for the yeast *Saccharomyces cerevisiae* [58]. For comparison purposes, it is not known how many generations, at the cell level, *D. septosporum* completes in a year.

Many confounding factors likely influence our estimated mutation rate. Firstly, the SNP mutation rates are probably over-estimated as the sets of isolates sampled at the earliest time point already had some genotypic diversity. Secondly, mutations may have occurred during storage, especially as the long-term storage method involved periodic regrowth to refresh the cultures and keep them viable. Thirdly, the mutation rate estimate was calculated using natural pathogen isolates in a commercial forest setting which, to the best of our knowledge, has not been reported previously. Due to differences in selection pressures, it is possible that mutation rates may differ between controlled laboratory and complex forest environments. Finally, published genome-wide estimates of SNPs are reliant on the accuracy of the assembly and variant calling software; a study to find SNP markers in a *Pinus radiata* transcriptome showed a 10-fold variation in SNP predictions depending on which of nine software combinations were used [59]. Despite these potential confounders, our data clearly indicate that natural *D. septosporum* isolates have become markedly more diverse over a 50-year period in the forest environment. 

### 4.3. Spore Germination, Growth Rates, and Copper Effects

The success of a fungal pathogen can be influenced by its rate of growth, the number of spores it releases and the ability of those spores to germinate. The 1960s isolates showed lower spore germination rates than the 1990s and 2000s isolates. Likewise, they showed a slightly lower, but nonsignificant, rate of growth in culture. While these characteristics could simply reflect their older age, they run counter to the increased virulence observed in the 1960s isolates as measured by increased fungal biomass in disease lesions. A possible explanation for their lower spore germination and growth rate in culture is inhibition by the high levels of dothistromin in the 1960s isolates. Dothistromin is toxic to a wide range of eukaryotes and prokaryotes, including fungi [60]. Its precise mode of action is unknown, but the harmful reactive oxygen species superoxide (O_2_^−^) and H_2_O_2_ are produced by reductive activation of dothistromin [61]. It is not known how and to what extent *D. septosporum* is resistant to its own toxin, but some detrimental effects might be expected from very high levels of dothistromin. This would suggest a natural upper limit to dothistromin levels, with a trade-off between the fitness of strains and their virulence.

No evidence was found to suggest that copper fungicide treatments over 50 years in New Zealand forests have selected for increased copper tolerance in *D. septosporum*. However, further experiments using a wider range of forms and concentrations of copper compounds are needed to conclusively show that no such adaptation has occurred. Given that copper fungicide sprays are only used at most every 2–3 years in New Zealand pine forests, and only when certain thresholds of disease symptoms are exceeded [10], the selection pressure exerted by these antifungal compounds in the forest environment may be minimal compared to frequently-sprayed agricultural crops.

### 4.4. Virulence and Dothistromin Production Decreased Over Time

All four isolates collected in the 1960s produced higher levels of the known virulence factor, dothistromin, than any of the isolates collected from the 1990s onwards. The effect of decade group on dothistromin levels was highly significant. This was unexpected as the secondary metabolism of fungal isolates often attenuates in culture [62] and levels of production might therefore be expected to be lower for the 1960s isolates, which had been in storage the longest. The specific effects of long-term storage on dothistromin production and virulence of *D. septosporum* are not known. But because even the ‘recent’ isolates had been in storage for 4–26 years, and subject to the same process of storage and refreshing as the 1960s isolates, storage effects are unlikely to account for the phenotypic differences seen between decade groups. 

While only a small number of historical isolates were available for this study, that three of the 1960s isolates have such high virulence is remarkable. Their increased virulence is concordant with their higher levels of dothistromin production in culture, compared to recent isolates, and these two phenotypes showed a strong positive correlation. Whether the production of dothistromin in culture reflects the capacity of an isolate to produce dothistromin in planta is an unanswered question; the small quantities of dothistromin produced in needle lesions make accurate assays difficult. However, because dothistromin is a known virulence factor [51] and virulence in planta is correlated with dothistromin levels in culture, our results suggest that high dothistromin producers in culture are also likely to be high dothistromin producers in planta. 

Notably, the 1960s isolate 16G had lower dothistromin levels and correspondingly lower virulence than the other 1960s isolates tested. 16G also had a lower sporulation rate and produced fewer disease lesions on pine needles compared to all other isolates tested (Table S4). This very low number of disease lesions skewed the mean disease lesion number for the 1960s decade group (Table 2 and Table S4). In pairwise SNP comparisons between all four 1960s isolates, 16G did not appear to be distinct; comparisons with 16G gave similar numbers of SNP differences as pairwise comparisons between the other (non-16G) isolates. Although specific differences were observed in certain genes (Table 3), we did not identify any specific polymorphisms in 16G that could easily account for the phenotypic differences from the other 1960s isolates, and 16G is not a close ancestor of all of the low virulence isolates sampled in later decades.

### 4.5. A Genetic Basis for Changes in Dothistromin Production and Virulence Could Not Be Determined

In our study, the significant differences in dothistromin production between 1960s and 2000s isolates could not be attributed to a genetic cause. The molecular basis of dothistromin biosynthesis and its regulation is well established [27,52,54], but no polymorphisms were found in the known biosynthetic or regulatory genes that could explain the patterns seen. Furthermore, no other specific polymorphisms associated with the different decade groups could be identified as correlating with this phenotype. However, the control of secondary metabolism is known to be complex and it is possible that gene expression differences or other factors are responsible for the differences in dothistromin levels. A recent study revealed the importance of chromatin modifications, including methylation of histones H3K9 and H3K27, and acetylation of H3K9, in the regulation of dothistromin biosynthesis [55]. In the potato blight pathogen *Phytophthora infestans*, differences in virulence between isolates of the pandemic clonal lineage EC-1 were associated with differences in gene expression, rather than genetic differences, and the authors proposed that epigenetic changes contributed to this phenotypic plasticity [63]. Intriguingly, the highest dothistromin producer 16F was shown to have a potential duplication of 1.2 Mb (76%) of chromosome 11. This mirrors a chromosome 11 duplication found in a very high dothistromin producing isolate from Germany (ALP3) [24], suggesting a possible influence of gene dosage on dothistromin levels.

### 4.6. Was There a Trade-Off between Virulence and Pathogen Transmission?

The higher dothistromin and virulence levels of the 1960s isolates, compared to all more recent isolates, suggest that changes to lower virulence occurred within the population following its introduction. Further work with additional isolates is needed to confirm this trend, although historical isolates are limited. Some studies of plant pathogen invasion and adaptation in agricultural crop plants have shown trends towards increased virulence over time [12]. In these cases, there is strong selection for increased virulence in crop hosts that are genetically and phenotypically homogeneous, replanted on an annual basis and subject to regular antifungal interventions [12,64]. *D. septosporum*, however, was likely already co-evolved to be virulent on its pine host prior to its introduction into New Zealand, in keeping with its most likely origin from other *Pinus* spp. hosts [22]. Furthermore, the long rotation times of forest crops might be expected to select, at least in the longer term, for pathogen isolates that are not so aggressive that they kill their hosts. This could occur as a trade-off between pathogen virulence and transmission, in which highly aggressive pathogens that kill their hosts consequently have fewer opportunities to find a suitable new host [8], even in the crowded plantation environment. Alternatively, there may be a trade-off between virulence and reproduction, because high virulence and/or high levels of dothistromin production might come at a cost to the pathogen by using up resources that could otherwise be devoted to growth and sporulation. 

Whether the reduced virulence seen over time in our study reflects reduced virulence in the forest over five decades is not possible to assess. Many factors such as fungicide spraying regimes, tree genotypes, and tree stocking rates, compounded by environmental influences, affect disease levels observed in the forest. While there appears to be a trend to more spraying for Dothistroma control over time (Figure S3), spray costs have reduced, prompting growers to spray at lower disease thresholds. Forest health surveillance started in New Zealand plantation forests in 1955. However, the data collected are of limited value for determining long term trends because surveyors were instructed to record only significant forest health problems. This introduced bias because sites where disease was absent or negligible were not included in mean annual disease estimates. Aerial survey assessments and records of areas sprayed with fungicide better reflect annual disease variation because means were calculated using assessments from all sites (Figure S3) but unfortunately long-term data are not available from these studies.

Despite the lack of long-term forest health data, local selection pressures may have shaped changes in the *D. septosporum* population after its arrival in New Zealand. Pine needles are a biotically rich environment, containing diverse endophytes and latent pathogens that are potential competitors [65,66]. Co-infection with different communities of commensal, mutualistic, or parasitic microorganisms can promote or temper the evolution of virulence [67,68] and these communities are likely to be different in a new environment, such as New Zealand. Elsewhere, and for other fungal pathogens, virulence has been attenuated by mycoviruses in the chestnut blight pathogen *Cryphonectria parasitica* [69] and by bacteria derived from a wheat microbiome in the wheat pathogen *Fusarium graminearum* [70]. It is possible that similar entities might have affected the virulence of New Zealand *D. septosporum* after its arrival. However, it is also possible that the reduction in dothistromin levels was a response to reduce auto-toxicity from this broad-spectrum toxin to *D. septosporum* itself, with the lower virulence to pines merely a pleiotropic effect of the lower toxin levels. Regardless, *D. septosporum* appears to have changed over time in the New Zealand forest environment despite its near clonal introduction event. 

## 5. Conclusions

Analysis of clonal isolates of *D. septosporum* collected over a 50-year period in New Zealand provided a unique opportunity to assess evolutionary change in an introduced forest pathogen over time. Genome diversity suggests that a restricted founder group, but more than a single isolate, was introduced to New Zealand in the 1960s and that further diversification occurred over time. Strikingly, isolates collected in the 1960s showed significantly higher virulence, and higher production of the virulence factor dothistromin, relative to more recent isolates, although additional analysis of historical isolates would be helpful to confirm these trends. We know of no other precedent for reduced virulence of a forest pathogen over time, but our results point to this trend for *D. septosporum*, perhaps in part as a response to maintaining the viability of its long-lived host. These changes were not coupled with responses to copper, the typical antifungal spray used over this period. This finding has implications for global forest health, suggesting the potential for incursions of highly virulent forest pathogens to become less virulent over time, regardless of human intervention. Further, the implications for practical resistance breeding are that, in some situations, even low levels of resistance or tolerance might be sufficient to improve the long-term health of trees.

## Figures and Tables

**Table 1:** *Dothistroma septosporum* isolates and genome mapping statistics.

Isolate ^a^	Year of Collection	*Pinus* Host Species	Region ^b^	Mapped Reads ^c^	% Mapped Reads ^c^	SRA Accession Number ^d^	Number of SNPs ^e^
NZE10	2005	*P. radiata*	Westland SI	n/a	n/a	PRJNA74753	n/a
16F	1965	*P. contorta*	Taupo	48490708	94.2	SRX3493225	227
16G	1967	*P. menziensii*	Taupo	43651635	93.1	SRX3493226	274
16N *	1969	*P. radiata*	Bay of Plenty	46614557	94.5	SRX3493227	311
16P *	1969	*P. radiata*	Bay of Plenty	46230530	95.1	SRX3493228	279
16R	1991	*P. radiata*	Waikato	51420587	95.3	SRX3493230	356
16V	1994	*P. radiata*	Bay of Plenty	49590713	94.9	SRX3493229	350
2737 *	2006	*P. radiata*	Taupo	46549085	93.8	SRX3493231	335
2738 *	2006	*P. radiata*	Taupo	45942540	94.4	SRX3493232	343
3287	2007	*P. radiata*	Taupo	47062342	93.9	SRX3493234	424
3769	2013	*P. radiata*	Gisborne	46616890	93.9	SRX3493233	362
4554 ^f^	2017	*P. yunnanensis*	Bay of Plenty	n/a	n/a	n/a	n/a

^a^ Scion (New Zealand Forest Research) collection number. * Indicates isolates collected on the same day (see text). ^b^ All isolates were collected from central regions of the North Island of New Zealand, except NZE10, which was from the west coast of the South Island (SI). ^c^ Number and % of reads mapped to the NZE10 reference genome (http://genome.jgi.doe.gov/Dotse1/). ^d^ Sequence Read Archive (SRA); Project accession number PRJNA426106 at http://www.ncbi.nlm.nih.gov. ^e^ Number of SNPs compared to the NZE10 genome, filtered to exclude variants in indel and repeat regions. ^f^ Isolate not sequenced; only used for dothistromin assays (n/a: not applicable).

**Table 2:** Summary of *Dothistroma septosporum* isolate phenotypes by decade group.

Phenotype ^a^	Decade Group (Mean ± SD) ^b^			All Groups Comparison ^c^		1960s vs. 2000s Comparison ^c^	
	1960s	1990s	2000s	*ω* ^2^	*p* Value	*ω* ^2^	*p* Value
Dothistromin ng/mg DW	54.10 ± 21.65	5.22 ± 2.07	10.62 ± 3.54	0.74	**0.002**	0.70	**0.003**
Growth 1 (mm/day) 0 Cu	0.61 ± 0.08	0.70 ± 0.17	0.69 ± 0.05	0.18	0.393	0.31	0.090
Growth 1 (mm/day) 50 Cu	0.56 ± 0.07	0.54 ± 0.10	0.57 ± 0.04	0.01	0.858	0.01	0.698
Growth 1 ratio 50/0 Cu	0.93 ± 0.06	0.79 ± 0.16	0.80 ± 0.07	0.33	**0.018**	0.49	**0.003**
Growth 2 (mm/day) 0 Cu	0.67 ± 0.09	0.77 ± 0.06	0.74 ± 0.12	0.14	0.434	0.11	0.322
Growth 2 (mm/day) 100 Cu	0.53 ± 0.04	0.60 ± 0.06	0.62 ± 0.05	0.41	**0.033**	0.47	**0.006**
Growth 2 ratio 100/0 Cu	0.81 ± 0.10	0.80 ± 0.13	0.84 ± 0.14	0.04	0.747	0.03	0.535
Sporulation × 10^6^/mL	5.25 ± 1.91	2.70 ± 1.56	5.31 ± 4.91	0.05	0.646	−0.01	0.979
% spore germination 0 Cu	35.79 ± 5.40	42.00 ± 11.15	44.52 ± 8.85	0.14	**0.014**	0.22	**0.008**
% spore germination 50 Cu	18.17 ± 5.66	13.33 ± 4.09	18.11 ± 5.77	0.05	0.187	−0.03	0.704
Spore germination ratio 50/0 Cu	0.49 ± 0.17	0.32 ± 0.11	0.43 ± 0.17	0.07	0.140	0.00	0.349
Disease lesions/100 needles	16.42 ± 10.39	21.33 ± 25.29	18.92 ± 14.52	−0.03	0.754	−0.01	0.558
Biomass in planta ng DNA/lesion	0.85 ± 0.20	0.55 ± 0.18	0.52 ± 0.09	0.50	**0.016**	0.55	**0.009**
Biomass in planta ng DNA/mg DW	1.51 ± 0.54	0.84 ± 0.14	0.82 ± 0.30	0.43	0.061	0.41	**0.040**

^a^ Growth 1 and 2 were two independent experiments; Cu was CuSO_4_·5H_2_O with concentrations in µg/mL; the percentage of spores that germinated was determined after 48 h incubation. ^b^ Values are mean ± SD of the replicates (3 ≥ *n* ≥ 6) for isolates within the decade group; see Table S4 for details. ^c^ Results of nested ANOVA and effect size analysis comparing three decade groups: 1960s (*n* = 4), 1990s (*n* = 2), 2000s (*n* = 5) or two decade groups: 1960s (*n* = 4), 2000s (*n* = 5). *p* values in bold indicate significance at *p* < 0.05.

**Table 3:** Genes with single nucleotide polymorphisms (SNPs) in more than one isolate.

Gene	16F	16G	16N	16P	16R	16V	2737	2738	3287	3769	NZE10	Predicted Function
*29186*	SN		SN	SN			SN					Glycoside hydrolase 72
*33501*								SN		SN		ATP binding
*34615*						SN		SN		SN		Not known
*38669*						R113Q		R113Q	R113Q	R113Q		Not known
*54626*	G579V				G579V							transporter
*71301*	SN		SN	SN			SN					ER protein
*71790*			G237D				G237D					Chromatin remodelling
*73448*	I658V		I658V	I658V			I658V					Ubiquitin ligase
*74128*			A17G				A17G					Nucleoside metabolism
*74795*	T482I		T482I				T482I					Vesicular transport
*79913*						Q212P		Q212P	Q212P	Q212P		Lipid metabolism
*71010*			I493F		R665W			FS 329			C570Y	Transcription regulation
*72963*		P456L				V728M						Chromatin remodelling
*74323*	C319*	E643G			FS144		S636*	FS 48	FS 547			RNA splicing
*137952*						C92R			I222M, N226H	L327R, S328P		RNA processing

Each row defines SNPs found in the indicated gene (JGI protein ID number) with amino acid sequence changes indicated, relative to NZE10. One SNP is shown for NZE10, which was different from all other isolates at that site. SNPs referred to as ‘SN’ are synonymous; FS are frameshifts with the corresponding amino acid position of the stop codon indicated; * indicates a nonsense mutation at the given site. Empty boxes indicate that a SNP was not found. Genes expressed in planta in NZE10 [27] are in bold and shaded dark gray when observed with >100 reads per million per kilobase (RPMK), mid gray >50 RPMK and light gray >10 RPMK; unshaded genes were not expressed. The predicted functions are from GO and KOG analyses. The genes in the top 11 rows had common SNPs in two or more isolates, genes in the bottom rows are expressed genes with independent mutations in two or more isolates.

## Supplementary Materials

The following are available online at https://www.mdpi.com/2076-2607/7/10/420/s1, Figure S1: Phylogeny of ten New Zealand and five global isolates of *Dothistroma septosporum,* Figure S2: Evidence for duplication and deletion in *Dothistroma septosporum* genomes, Figure S3: Assessing long term trends in Dothistroma needle blight severity in New Zealand forests, Table S1: Single Nucleotide Polymorphisms in the *D. septosporum* genomes, Table S2: Mutation rates, Table S3: Nested ANOVA and effect size analysis to test the null hypothesis of no effect of decade group on phenotype, Table S4: Phenotype data for *D. septosporum* isolates, Table S5: *D. septosporum* NZE10 curated repeats over 200 bp.
