# Supplementary material for: Reduced Virulence of an Introduced Forest Pathogen over 50 Years
Source: Microorganisms. 2019 Oct 5;7(10):420. doi: 10.3390/microorganisms7100420 (PMC6843257; doi:10.3390/microorganisms7100420)

Fig. S1 Phylogeny of ten New Zealand isolates and five global isolates of *D. septosporum*

The neighbour-joining phylogeny was prepared with whole-genome filtered SNPs (compared to *D. septosporum* NZE10). The New Zealand isolates are grouped in a clade distinct from isolates from five other countries (COL Colombia, ECU Ecuador, CAN Canada, ALP3 Germany, RUS1 Russia; Bradshaw et al 2019 Molecular Plant Pathology 20: 784-799) and there is much lower genetic diversity between the New Zealand isolates than between the 'other country' isolates. Numbers represent percentages of 5000 bootstrap replications.

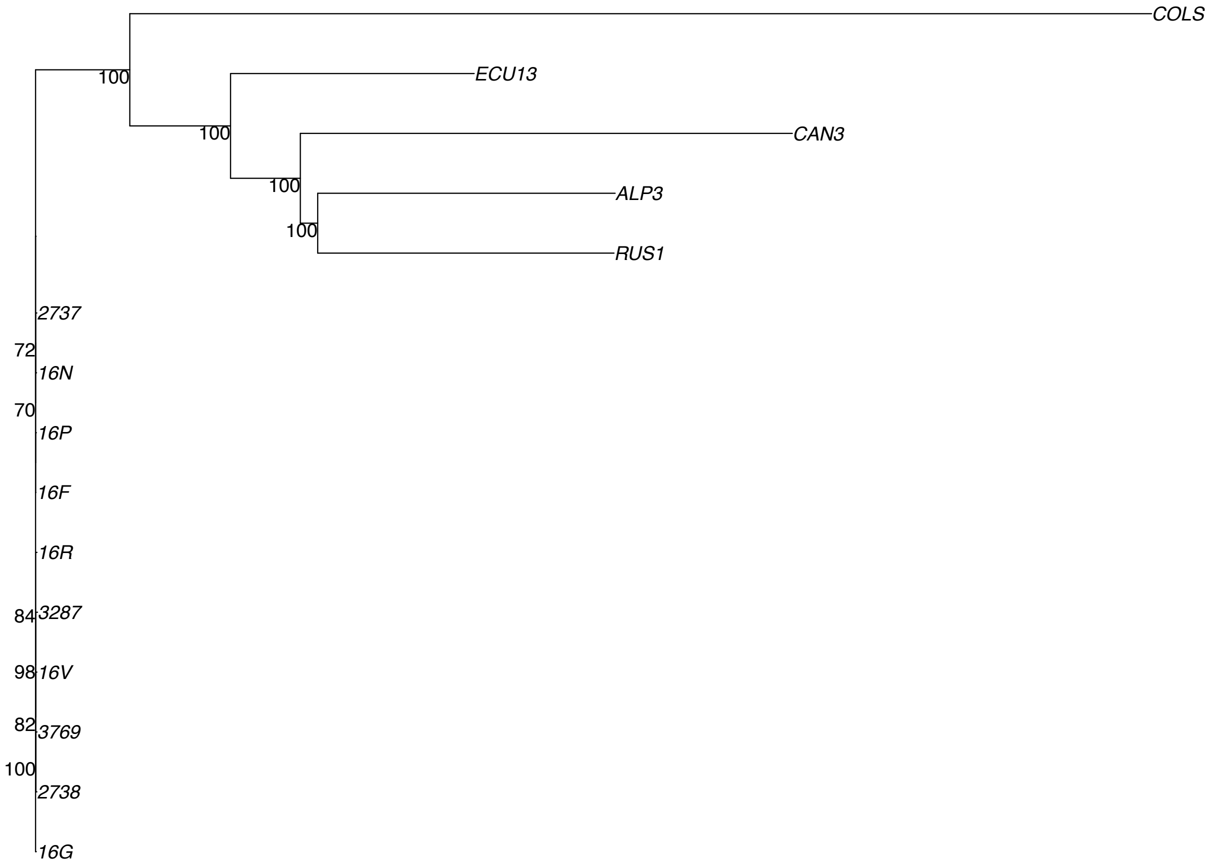

Supplement: Supplementary file 1 [file microorganisms-07-00420-s001.zip › Figure S1.pdf]
