# Supplementary material for: Reduced Virulence of an Introduced Forest Pathogen over 50 Years
Source: Microorganisms. 2019 Oct 5;7(10):420. doi: 10.3390/microorganisms7100420 (PMC6843257; doi:10.3390/microorganisms7100420)
Supplement: Supplementary file 1 [file microorganisms-07-00420-s001.zip › Figure S2.pdf]

Supporting Information Figure S2

Evidence for duplication and deletion in *D. septosporum* genomes

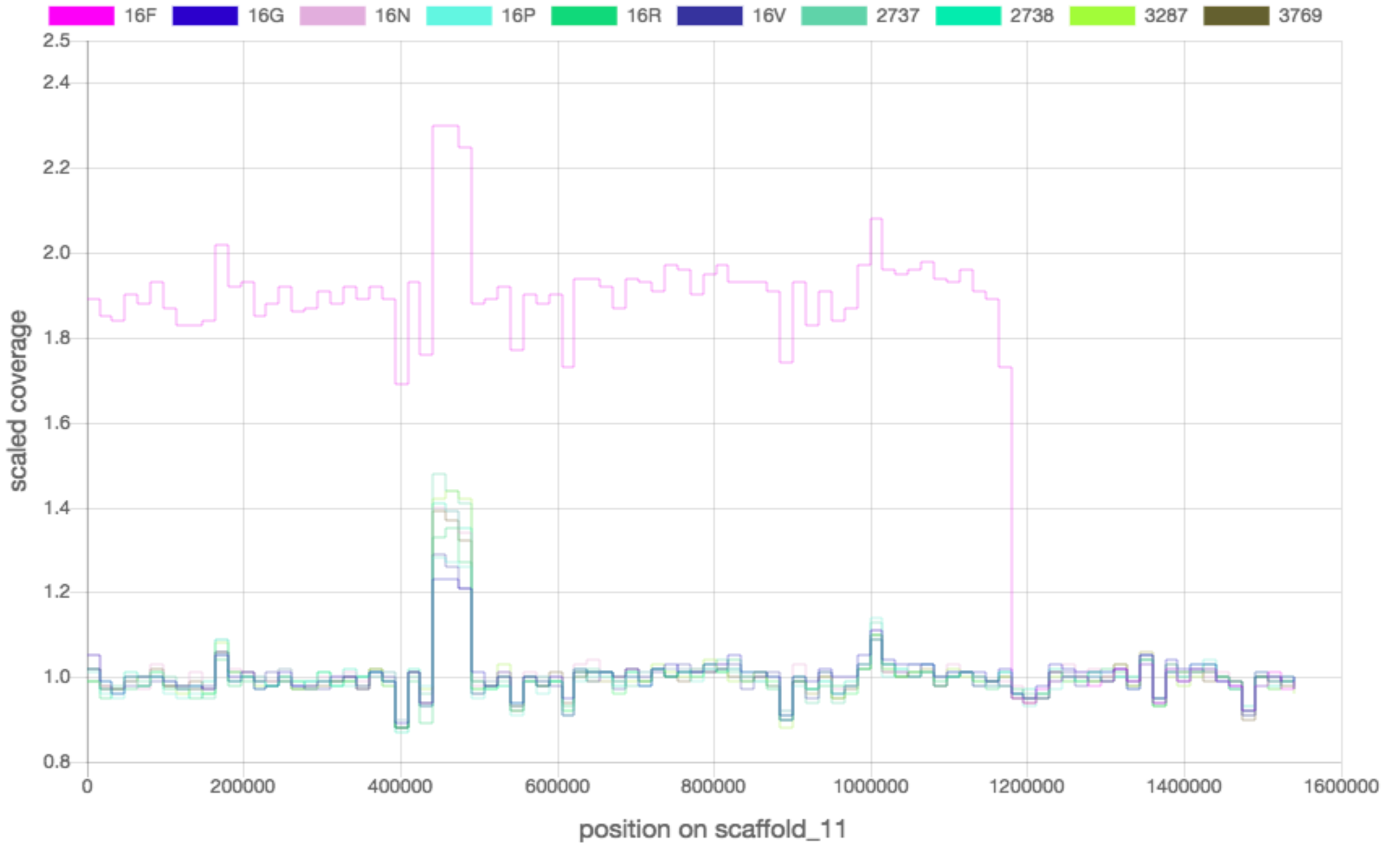

(a) Scaled read coverage of all isolates for chromosome 11 suggested that 16F has a duplication.

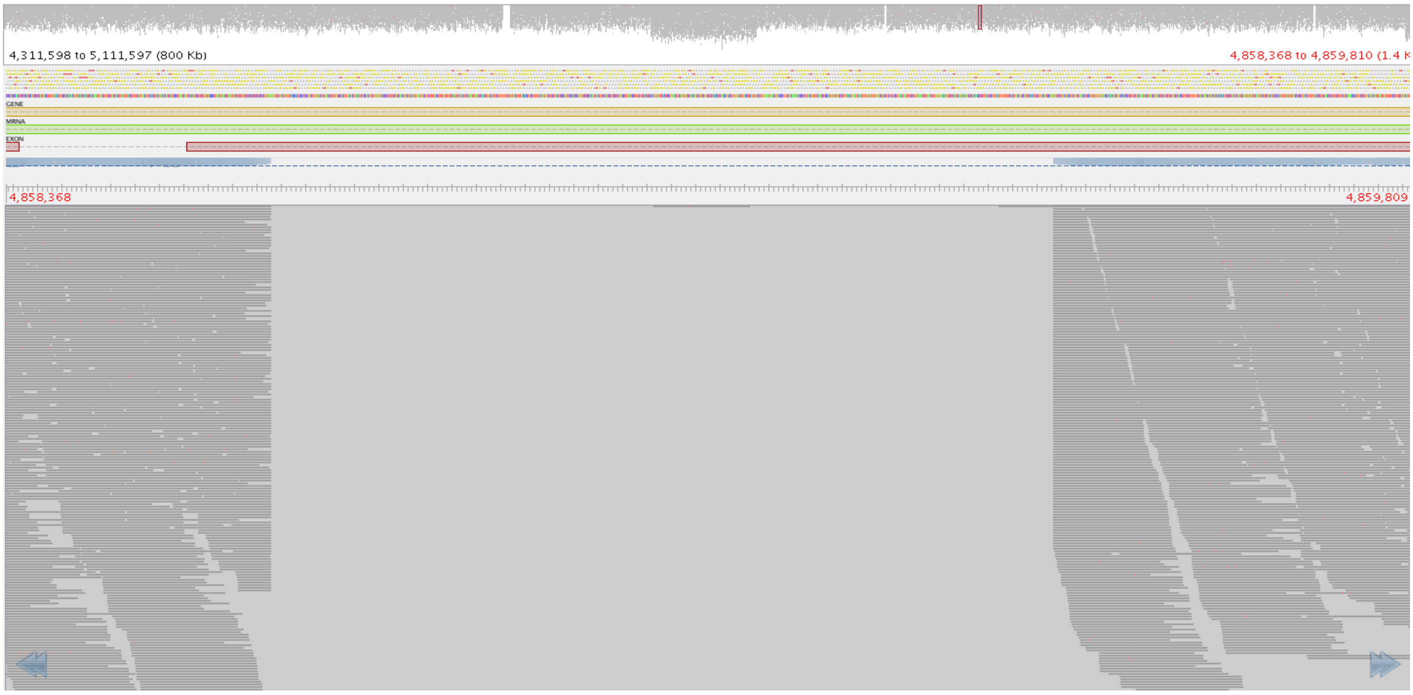

(b) A suspected deletion, found in isolates 2738 and 3769. Reads on either side of the suspected deletion are paired, providing strong evidence for the existence of a deletion. The deletion is approximately 1000 bases in length and falls within a gene encoding a hypothetical protein (protein ID 68999) on chromosome 1 (4858500-4859438).
