# Supplementary material for: Reduced Virulence of an Introduced Forest Pathogen over 50 Years
Source: Microorganisms. 2019 Oct 5;7(10):420. doi: 10.3390/microorganisms7100420 (PMC6843257; doi:10.3390/microorganisms7100420)
Supplement: Supplementary file 1 [file microorganisms-07-00420-s001.zip › Figure S3.pdf]

Figure S3

Assessing long term trends in Dothistroma needle blight severity in New Zealand forests

(a)

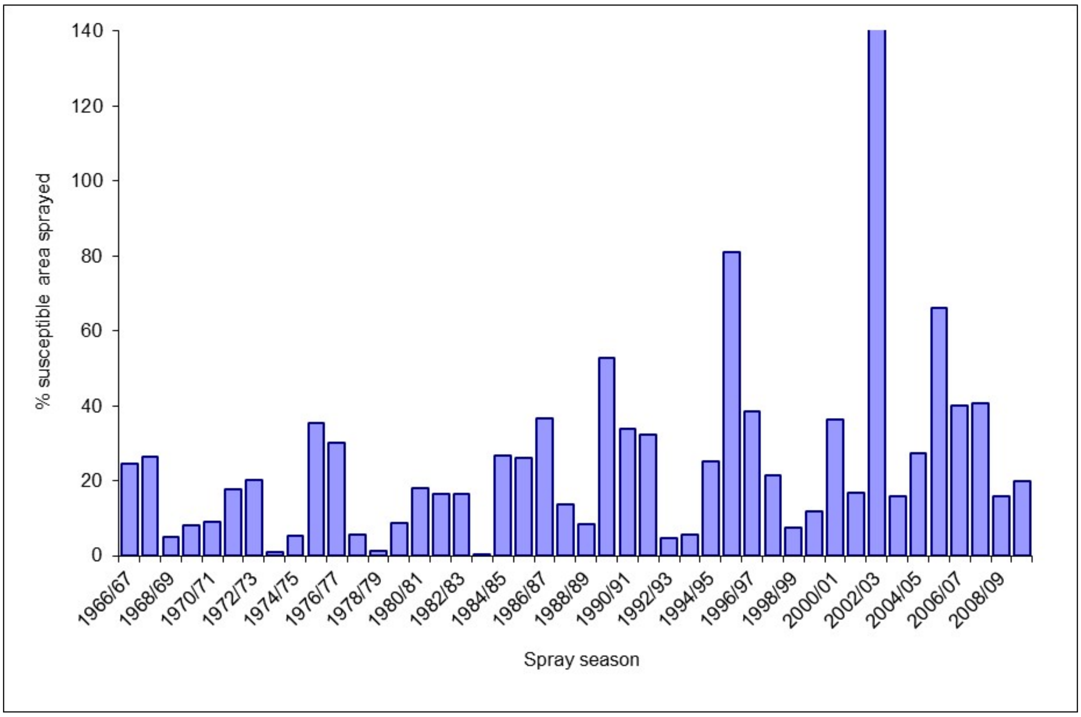

(b)

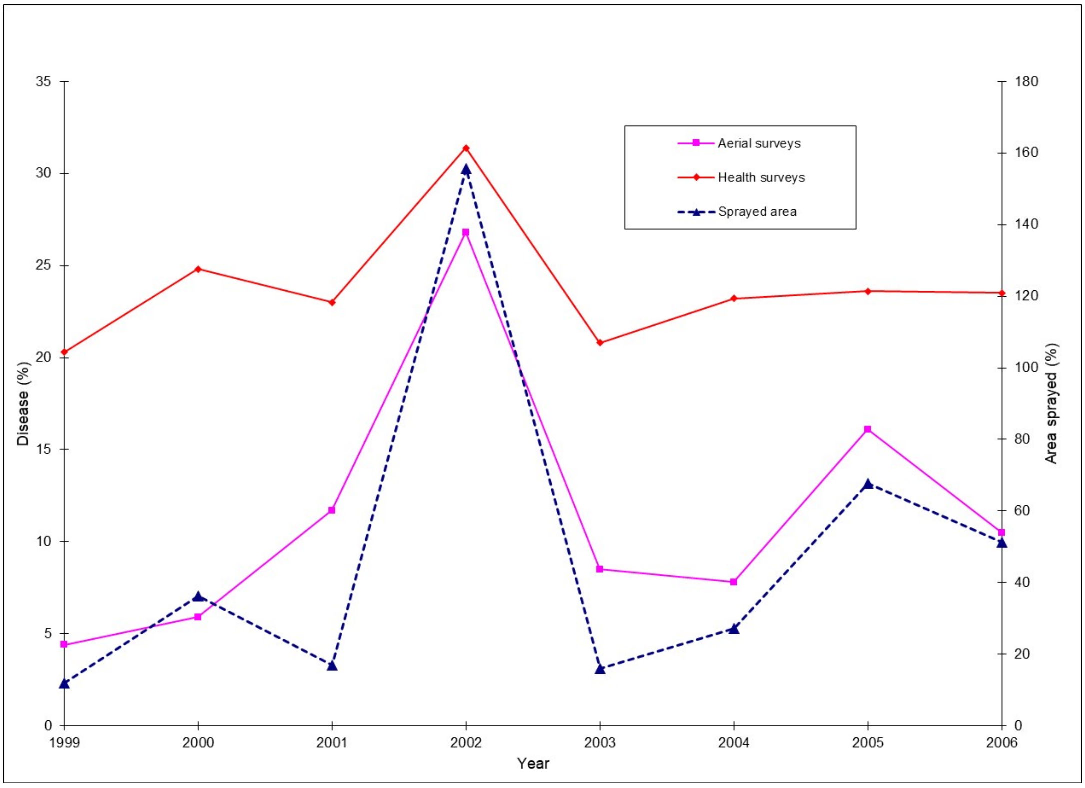

(a) The percentage of susceptible *Pinus radiata* area (aged 1-15 years) sprayed in the central North Island of New Zealand from 1966 to 2009.

(b) Three metrics for estimating Dothistroma needle blight severity are shown. Data from forest health surveys include bias due to a policy of only recording observations when disease severity exceeded 20%. Targeted aerial surveys and area sprayed demonstrate better reflect annual disease variation because means were calculated using assessments from all sites. Unfortunately long term data are not available.
