# Supplementary material for: Reduced Virulence of an Introduced Forest Pathogen over 50 Years
Source: Microorganisms. 2019 Oct 5;7(10):420. doi: 10.3390/microorganisms7100420 (PMC6843257; doi:10.3390/microorganisms7100420)
Supplement: Supplementary file 1 [file microorganisms-07-00420-s001.zip › Table_S1.docx]

## **Table S1 Single Nucleotide Polymorphisms in the *Dothistroma septosporum* genomes**

|  | **Whole genome SNP numbers**^†^ | | | | **In-gene SNP numbers** | | | | |
| --- | --- | --- | --- | --- | --- | --- | --- | --- | --- |
| **Isolate** | **Total** | indels removed | indels removed, repeats masked^‡^ | **Total** | | indels  removed, repeats masked^‡^ | **non-synonymous** indels  removed, repeats masked^‡^ | **synonymous** indels  removed, repeats masked^‡^ |  |
| **16F** | 3053 | 2667 | 227 | 460 | | 75 | 38 | 19 |  |
| **16G** | 3363 | 2940 | 274 | 462 | | 78 | 37 | 20 |  |
| **16N** | 3552 | 3081 | 311 | 497 | | 96 | 49 | 23 |  |
| **16P** | 3285 | 2888 | 279 | 477 | | 89 | 45 | 21 |  |
| **16R** | 3874 | 3381 | 356 | 522 | | 101 | 50 | 22 |  |
| **16V** | 3725 | 3237 | 350 | 516 | | 92 | 44 | 22 |  |
| **2737** | 3701 | 3245 | 335 | 508 | | 99 | 49 | 21 |  |
| **2738** | 3701 | 3237 | 343 | 513 | | 96 | 43 | 24 |  |
| **3287** | 4350 | 3741 | 424 | 561 | | 109 | 48 | 26 |  |
| **3769** | 3730 | 3237 | 362 | 529 | | 98 | 51 | 25 |  |

^†^ All Single Nucleotide Polymorphisms (SNPs) are relative to the reference genome of *D. septosporum* NZE10.

^‡^ Indels removed and SNPs falling within repeat regions removed according to Methods.
