# Supplementary material for: Reduced Virulence of an Introduced Forest Pathogen over 50 Years
Source: Microorganisms. 2019 Oct 5;7(10):420. doi: 10.3390/microorganisms7100420 (PMC6843257; doi:10.3390/microorganisms7100420)
Supplement: Supplementary file 1 [file microorganisms-07-00420-s001.zip › Table_S3.docx]

**Table S3. Nested ANOVA and effect size analysis to test the null hypothesis of no effect of decade group on phenotype**

|  | **Analysis 1*: 1960s – 1990s – 2000s** | | | | | **Analysis 2: 1960s –2000s** | | | | |
| --- | --- | --- | --- | --- | --- | --- | --- | --- | --- | --- |
| **Variable (phenotype)** | **Df** | **F** | **P** | **sig**^†^ | *ω***^2^**^‡^ | **Df** | **F** | **P** | **sig** | *ω***^2^** |
| Dothistromin ng/mg DW | 2, 8 | 14.87 | 0.0020 | ** | 0.7377 | 1, 7 | 21.09 | 0.0025 | ** | 0.6967 |
| Growth 1 (mm/day) 0 Cu | 2, 8 | 1.053 | 0.3926 |  | 0.1816 | 1, 7 | 3.875 | 0.0897 |  | 0.3055 |
| Growth 1 (mm/day) 50 Cu | 2, 8 | 0.1564 | 0.8578 |  | 0.0119 | 1, 7 | 0.1638 | 0.6978 |  | 0.0119 |
| Growth 1 ratio 50/0 Cu | 2, 8 | 6.891 | 0.0182 | * | 0.328 | 1, 7 | 20.06 | 0.0029 | ** | 0.4883 |
| Growth 2 (mm/day) 0 Cu | 2, 8 | 0.9294 | 0.4336 |  | 0.1426 | 1, 7 | 1.137 | 0.3216 |  | 0.1079 |
| Growth 2 (mm/day) 100 Cu | 2, 8 | 5.382 | 0.0330 | * | 0.4064 | 1, 7 | 15.61 | 0.0055 | ** | 0.4739 |
| Growth 2 ratio 100/0 Cu | 2, 8 | 0.3023 | 0.7472 |  | 0.0350 | 1, 7 | 0.4258 | 0.5349 |  | 0.0274 |
| Sporulation x10^6^/ml | 2, 8 | 0.4615 | 0.6461 |  | 0.0545 | 1, 7 | 0.00075 | 0.9789 |  | -0.0149 |
| % spore germination 0 Cu | 2, 8 | 7.708 | 0.0136 | * | 0.1352 | 1, 7 | 13.39 | 0.0081 | ** | 0.2233 |
| % spore germination 50 Cu | 2, 8 | 2.08 | 0.1873 |  | 0.0521 | 1, 7 | 0.1568 | 0.7039 |  | -0.0328 |
| Spore germination ratio 50/0 Cu | 2, 8 | 2.538 | 0.1401 |  | 0.0689 | 1, 7 | 1.009 | 0.3485 |  | -0.0034 |
| Disease lesions per 100 needles | 2, 8 | 0.2922 | 0.7543 |  | -0.0324 | 1, 7 | 0.3785 | 0.5579 |  | -0.0104 |
| Biomass *in planta* ng DNA / lesion | 2, 8 | 7.237 | 0.0161 | * | 0.4982 | 1, 7 | 12.94 | 0.0088 | ** | 0.5517 |
| Biomass *in planta* ng DNA / mg DW | 2, 8 | 4.037 | 0.0613 |  | 0.4281 | 1, 7 | 6.308 | 0.0403 | * | 0.4085 |

^*^All eleven isolates (including NZE10) were included in analysis 1, but only the four 1960s isolates & five 2000s isolates in analysis 2.

^†^ ** P<0.01; * P<0.05

^‡^*ω^2^* (omega^2^) is an unbiased estimator of effect size
